# Supplementary material for: Resolving cell state in iPSC-derived human neural samples with multiplexed fluorescence imaging
Source: Commun Biol. 2021 Jun 24;4:786. doi: 10.1038/s42003-021-02276-x (PMC8225800; doi:10.1038/s42003-021-02276-x)
Supplement: Supplementary file 2 — Supplementary Information [file 42003_2021_2276_MOESM2_ESM.pdf]

SUPPLEMENTARY INFORMATION

Resolving cell state in iPSC-derived human neural samples with multiplexed fluorescence imaging

Martin L. Tomov[1,2,3], Alison O'Neil[1.2], Hamdah S. Abbasi[4], Beth A. Cimini[4], Anne E. Carpenter[4], Lee L. Rubin[1.2]*, Mark Bathe[1,3]*.

1. Stanley Center for Psychiatric Research, Broad Institute of MIT and Harvard, Cambridge, MA, USA
2. Dept. of Stem Cell and Regenerative Biology, Harvard University, Cambridge, MA, USA
3. Dept. of Biological Engineering, MIT, Cambridge, MA, USA
4. Imaging Platform at Broad Institute of MIT and Harvard, Cambridge, MA, USA

*These authors jointly supervised this work.

Correspondence to: Lee Rubin lee_rubin@harvard.edu and Mark Bathe mark.bathe@mit.edu

a

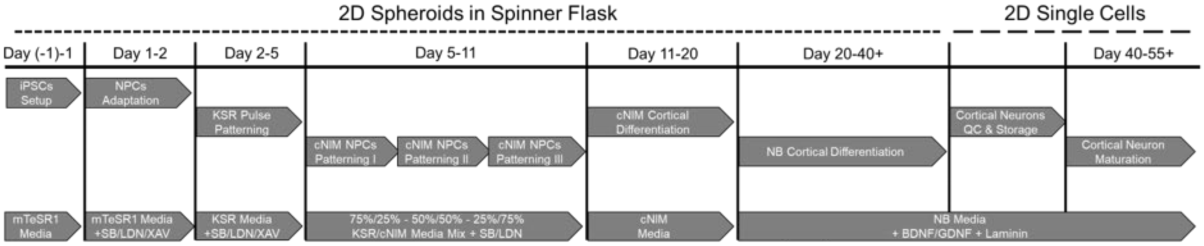

KSR Media	500mL	Full bottle
KO DMEM	405mL	509mL
20% Knock out serum	80mL	93.5mL
β-mercaptoethanol	0.5mL	625mL
100x NEAA	5mL	6.25mL
100x P/S	5mL	6.25mL
100x GlutaMAX	5mL	6.25mL

cNIM Media	500mL	Full bottle
DMEM: F12	476mL	509mL
Glucose (20% w/v)	4mL	4.2mL
100x NEAA	5mL	5.3mL
100x P/S	5mL	5.3mL
100x GlutaMAX	5mL	5.3mL
100x N2	5mL	5.3mL
50x B27 minus vitA	10mL	10.6mL

NB Media	500mL	Full bottle
Neurobasal	480mL	509mL
100x NEAA	5mL	5.3mL
100x P/S	5mL	5.3mL
100x GlutaMAX	5mL	5.3mL
100x N2	5mL	5.3mL
Ascorbic Acid (20mM)	5mL	5.3mL
50x B27 minus vitA	10mL	10.6mL

b

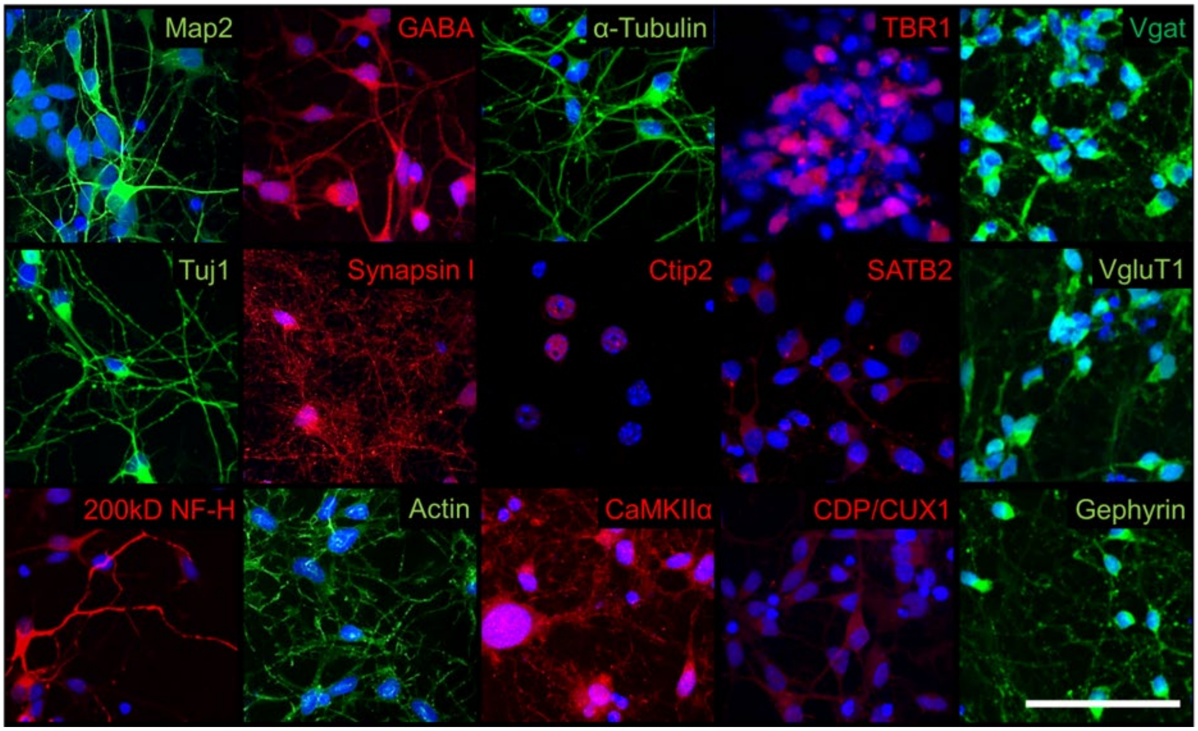

Supplementary Figure 1: Directed differentiation towards mature iPSC-derived cortical neurons, utilizing the spinner flask platform. (a) Schematic of cortical differentiation *in vitro*, with highlighted important milestones within the protocol. **(b)** A selection of markers used to characterize the cultures and confirm mature cortical commitment. Scale bar is 100 microns.

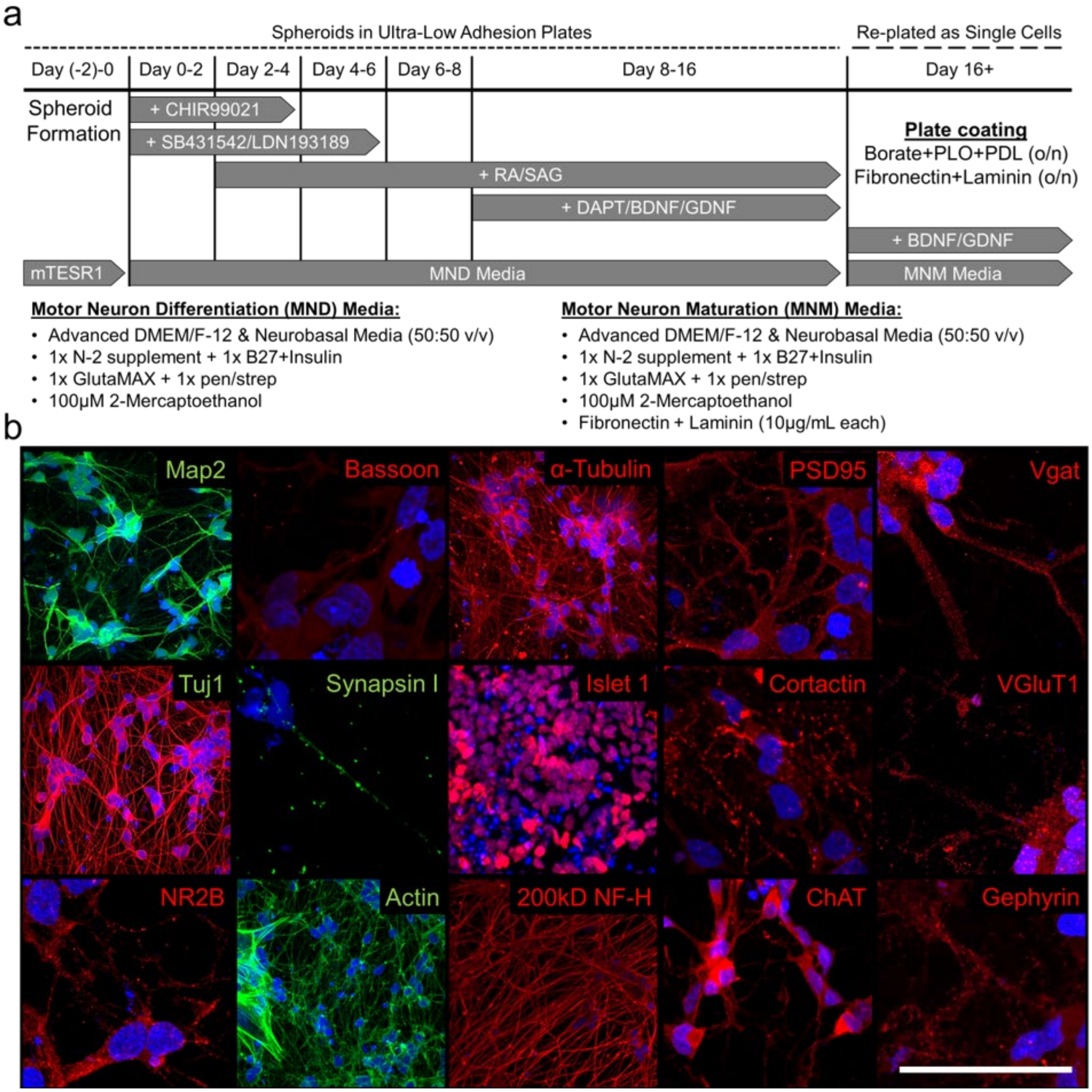

Supplementary Figure 2: Directed differentiation towards mature iPSC-derived motor neurons, utilizing the spinner flask platform. (a) Schematic of motor neuron differentiation *in vitro*, with highlighted important milestones within the protocol. **(b)** A selection of markers used to characterize the cultures and confirm motor neuron commitment. Scale bar is 50 microns.

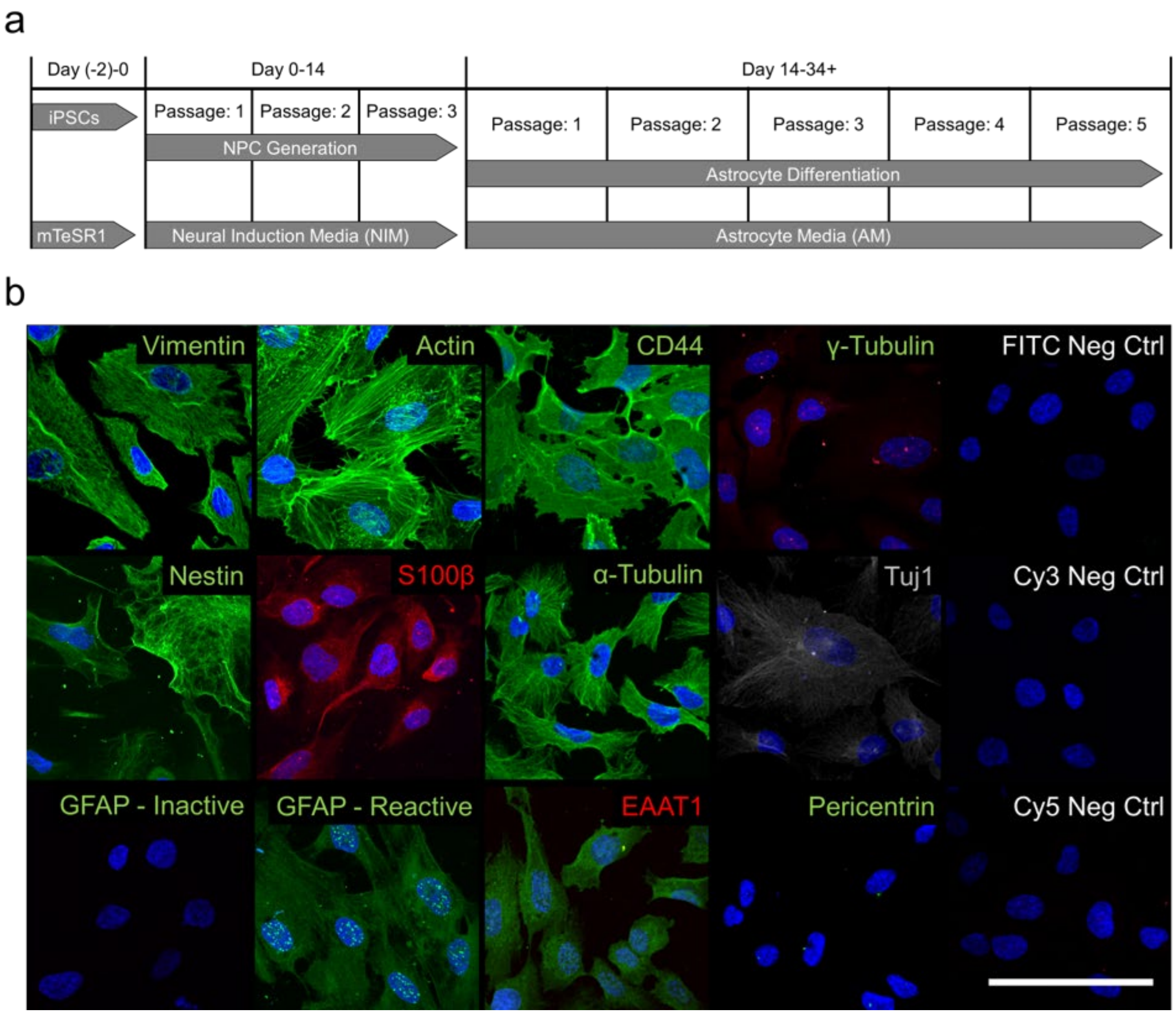

Supplementary Figure 3: Directed differentiation towards mature iPSC-derived astrocytes, utilizing the spinner flask platform. (a) Schematic of astrocyte differentiation *in vitro*, with highlighted important milestones within the protocol. **(b)** A selection of markers used to characterize the cultures and confirm astrocyte differentiation. Scale bar is 50 microns.

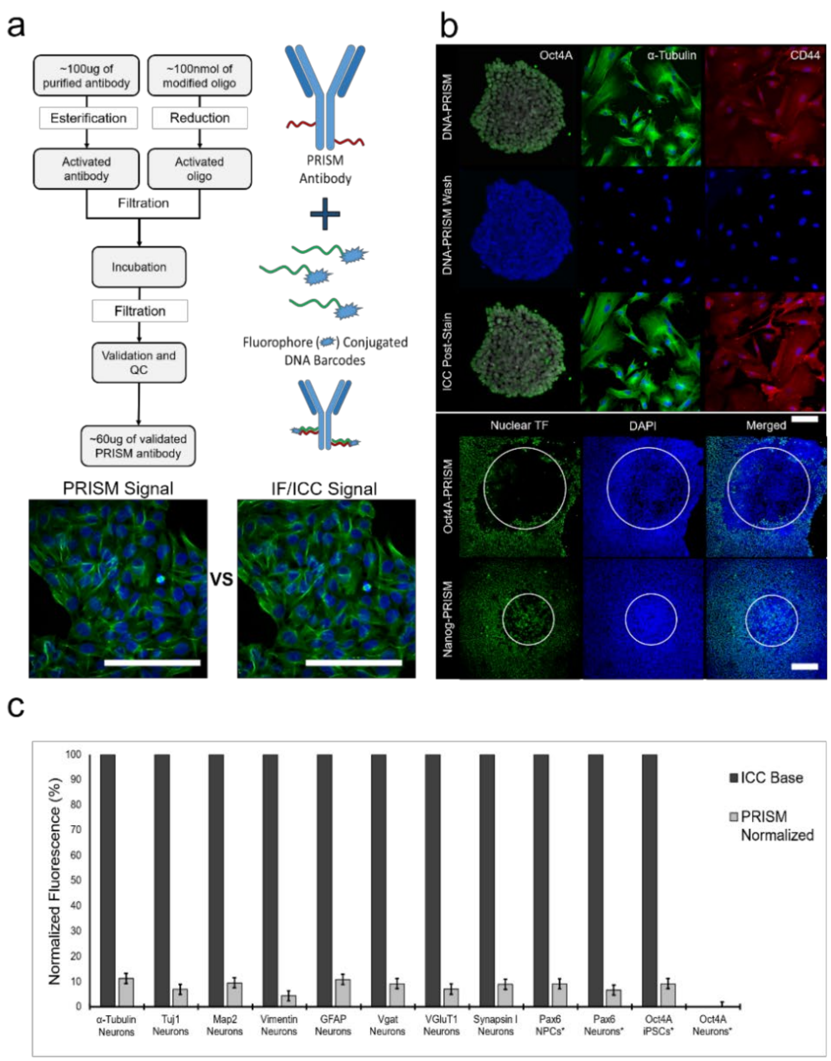

d

Marker	ICC Values Trial 1	ICC Values Trial 2	ICC Values Trial 3	PRISM Values Trial 1	PRISM Values Trial 2	PRISM Values Trial 3	Fold Difference	ICC Normalized	PRISM Normalized
α-Tubulin Neurons	29.15	33.49	27.10	3.28	2.97	4.10	8.67	100	11.54
Tuj1 Neurons	149.94	138.23	112.98	10.37	13.33	8.01	12.65	100	7.90
Map2 Neurons	17.97	14.02	16.00	1.71	1.49	1.83	9.54	100	10.48
Vimentin Neurons	67.81	65.11	69.08	2.99	2.78	3.72	21.29	100	4.70
GFAP Neurons	26.37	24.87	23.94	2.86	2.58	3.12	8.78	100	11.39
Vgat Neurons	56.16	51.98	62.85	5.16	5.33	5.84	10.47	100	9.55
VGluT1 Neurons	40.30	40.27	45.72	2.85	2.44	2.63	15.95	100	6.27
Synapsin I Neurons	42.82	41.09	48.63	3.83	3.22	4.01	11.98	100	8.35
Pax6 NPCs*	79.86	85.67	64.01	7.28	6.99	7.58	10.50	100	9.52
Pax6 Neurons*	1.43	1.23	1.11	0.10	0.09	0.12	12.33	100	8.11
Oct4A iPSCs*	0.69	0.59	0.64	0.06	0.03	0.06	12.52	100	7.99
Oct4A Neurons*	0.00	0.00	0.00	0.00	0.00	0.00	N/A	N/A	N/A

Supplementary Figure 4: Highlights of the generation and validation of high content PRISM antibody platform, compared to traditional IF/ICC fluorescence. (a) PRISM antibody generation of commercially available antibodies and PRISM-based IF/ICC data generation, including a representative comparison of α-tubulin fluorescent readouts. **(b)** PRISM validation by comparing to IF/ICC staining in three major cell compartments, including several stem cell nuclear transcription factors in pure pluripotent cultures (top) and in mixed pluripotent and differentiating cultures (bottom), where the Oct4A and Nanog signals are decreased or gone (circled). **(c)** Normalized fluorescence yield of PRISM markers compared to IF/ICC controls, normalized to 100% based on IF/ICC signal, in the same culture across the full validated marker panel (Standard Error of Mean shown; n=3). **(d)** Individual fluorescence points are shown in the table. Scale bars are 50 microns.

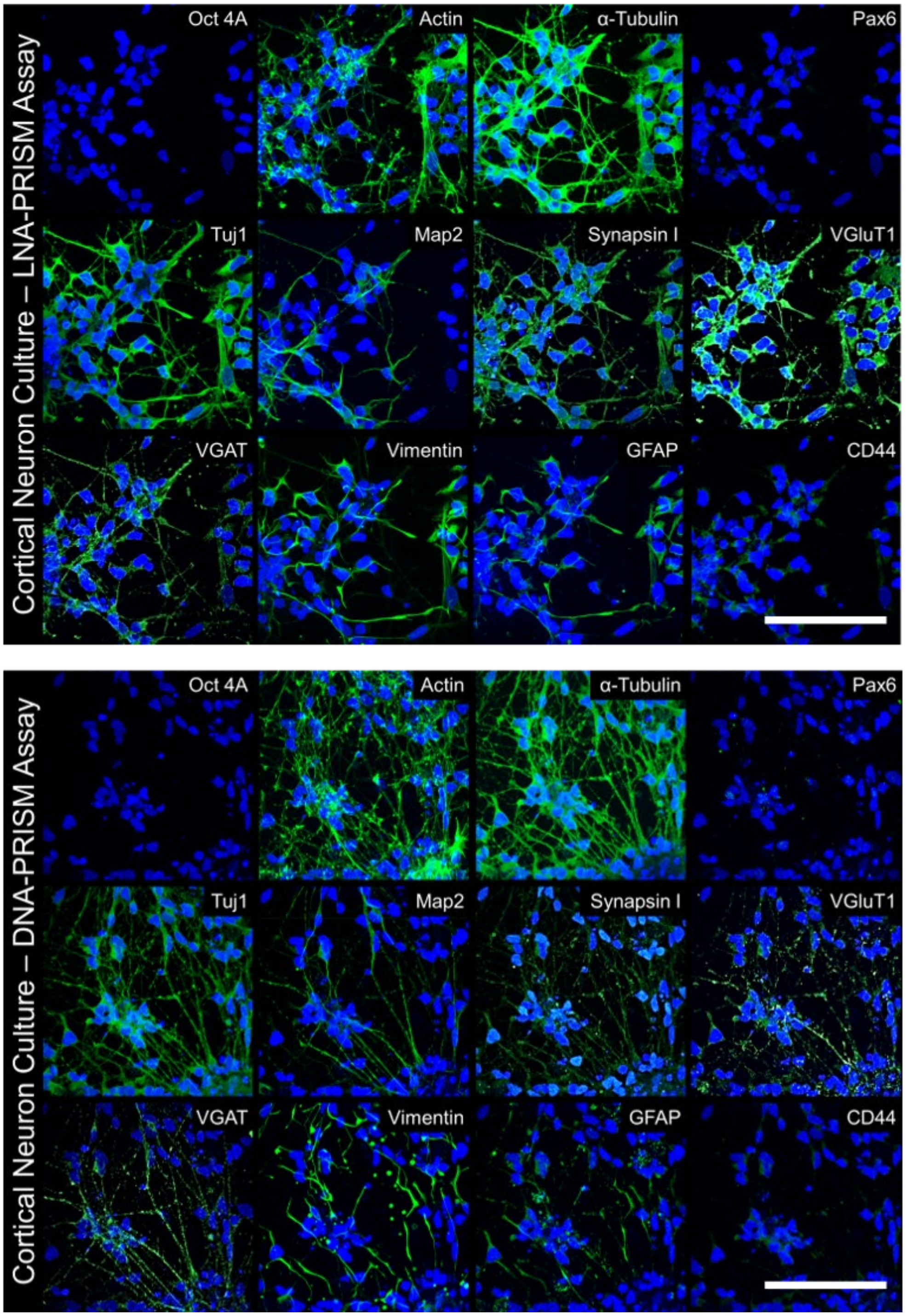

Supplementary Figure 5: Both LNA-PRISM and DNA-PRISM imager strands can generate high-content data in 2D dissociated human cortical neurons derived from iPSCs. Staining comparison between the LNA imager strand set and the same antibodies conjugated to work with the DNA imager strand set. Cells were counterstained with DAPI and Phalloidin488. The PRISM marker staining sequence was Tuj1 > α-Tubulin > Map2 > Synapsin I > Vimentin > VGluT1 > Pax6 > GFAP > VGAT > Oct4A > CD44 for both the DNA-PRISM and LNA-PRISM antibodies. Scale bars are 100 microns.

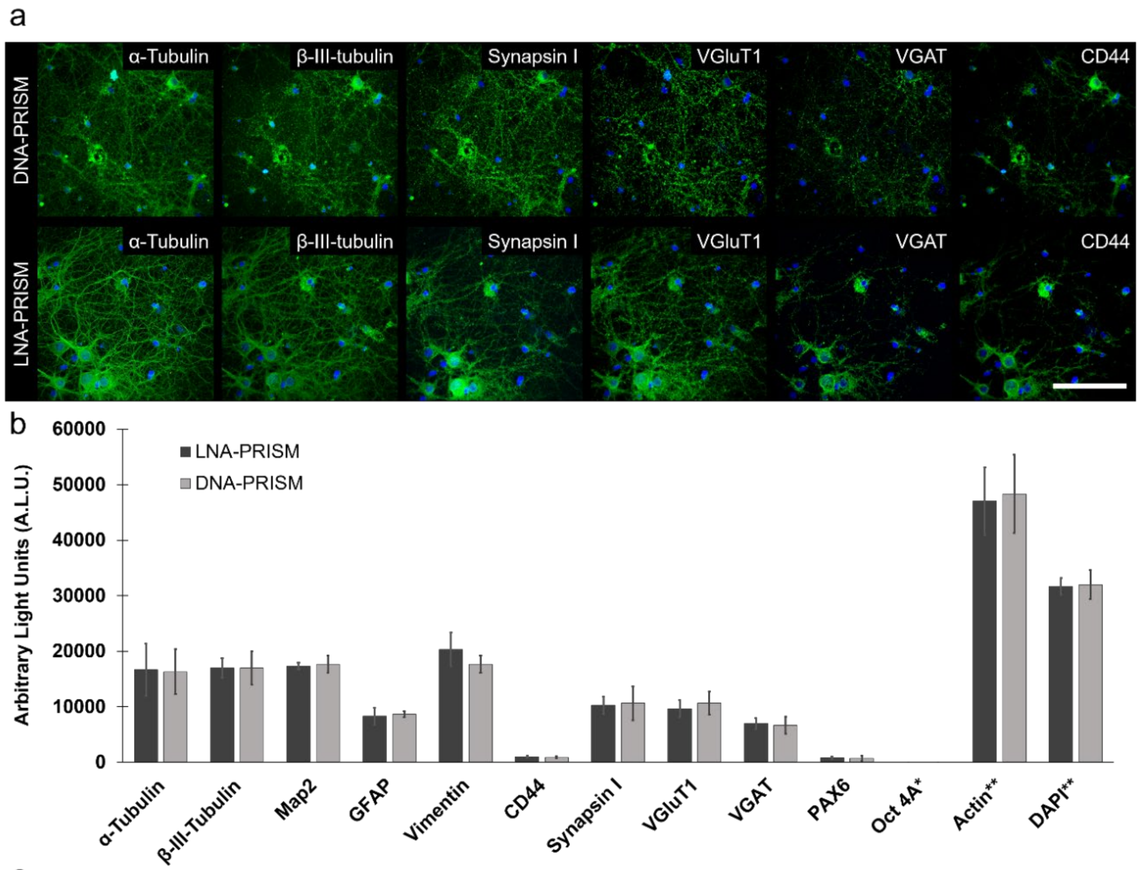

c

	ALU 1 (FIJI)		ALU 2 (FIJI)		ALU 3 (FIJI)		ALU Average (FIJI)			
Marker	LNA-PRISM	DNA-PRISM	LNA-PRISM	DNA-PRISM	LNA-PRISM	DNA-PRISM	LNA-PRISM	DNA-PRISM	Std.Dev. LNA	Std.Dev. DNA
α-Tubulin	22000	14000	13000	21000	15000	14000	16666.67	16333.33	4725.82	4041.45
β-III-Tubulin	19000	17000	16000	14000	16000	20000	17000.00	17000.00	1732.05	3000.00
Map2	17000	18000	17000	19000	18000	16000	17333.33	17666.67	577.35	1527.53
GFAP	8000	9000	7000	9000	10000	8000	8333.33	8666.67	1527.53	577.35
Vimentin	23000	16000	21000	19000	17000	18000	20333.33	17666.67	3055.05	1527.53
CD44	1000	900	1200	1100	900	700	1033.33	900.00	152.75	200.00
Synapsin I	10000	8000	9000	14000	12000	10000	10333.33	10666.67	1527.53	3055.05
VGluT1	8000	10000	10000	9000	11000	13000	9666.67	10666.67	1527.53	2081.67
VGAT	7000	8000	8000	7000	6000	5000	7000.00	6666.67	1000.00	1527.53
PAX6	600	400	800	600	1000	1200	800.00	733.33	200.00	416.33
Oct 4A*	0	0	0	0	0	0	0.00	0.00	0.00	0.00
Actin**	40000	41000	50000	49000	51000	55000	47000.00	48333.33	6082.76	7023.77
DAPI**	32000	31000	30000	30000	33000	35000	31666.67	32000.00	1527.53	2645.75

Supplementary Figure 6: LNA (Locked Nucleic Acid)-PRISM intensity compared to optimized DNA-PRISM intensity shows comparable fluorescence readouts based on the Arbitrary Light Units (A.L.U.) on the same microscope. Representative IF images of rat cortical neurons are shown in the top panel, covering the major cell areas that are used in this paper for cortical culture characterization. Signal strength comparisons between LNA-PRISM and DNA-PRISM imaging strands was done on three different confocal microscopes (ImageXpress, Molecular Devices; Opera Phenix, PerkinElmer; C2+ modified manual confocal system, NIKON) and at least three independent staining experiments. Oct 4A staining (*) was not detected since there are no pluripotent stem cells left at the time of culture evaluation in either differentiated human iPSC-derived cortical cultures, or in dissociated rat/mouse cortical cultures. Actin and nuclear staining (**) were done using commercially available reagents – Phalloidin488 for actin and DAPI for nuclear counter-staining. Bottom panel graph used A.L.U. values averaged across three independent experiments, done on the same microscope (Opera Phenix; PerkinElmer). Error bars show SEM values (no statisitically significant differences between the LNA and DNA signal for each pair of analogous stainings). Raw values are shown in the table. Scale bar is 100 microns.

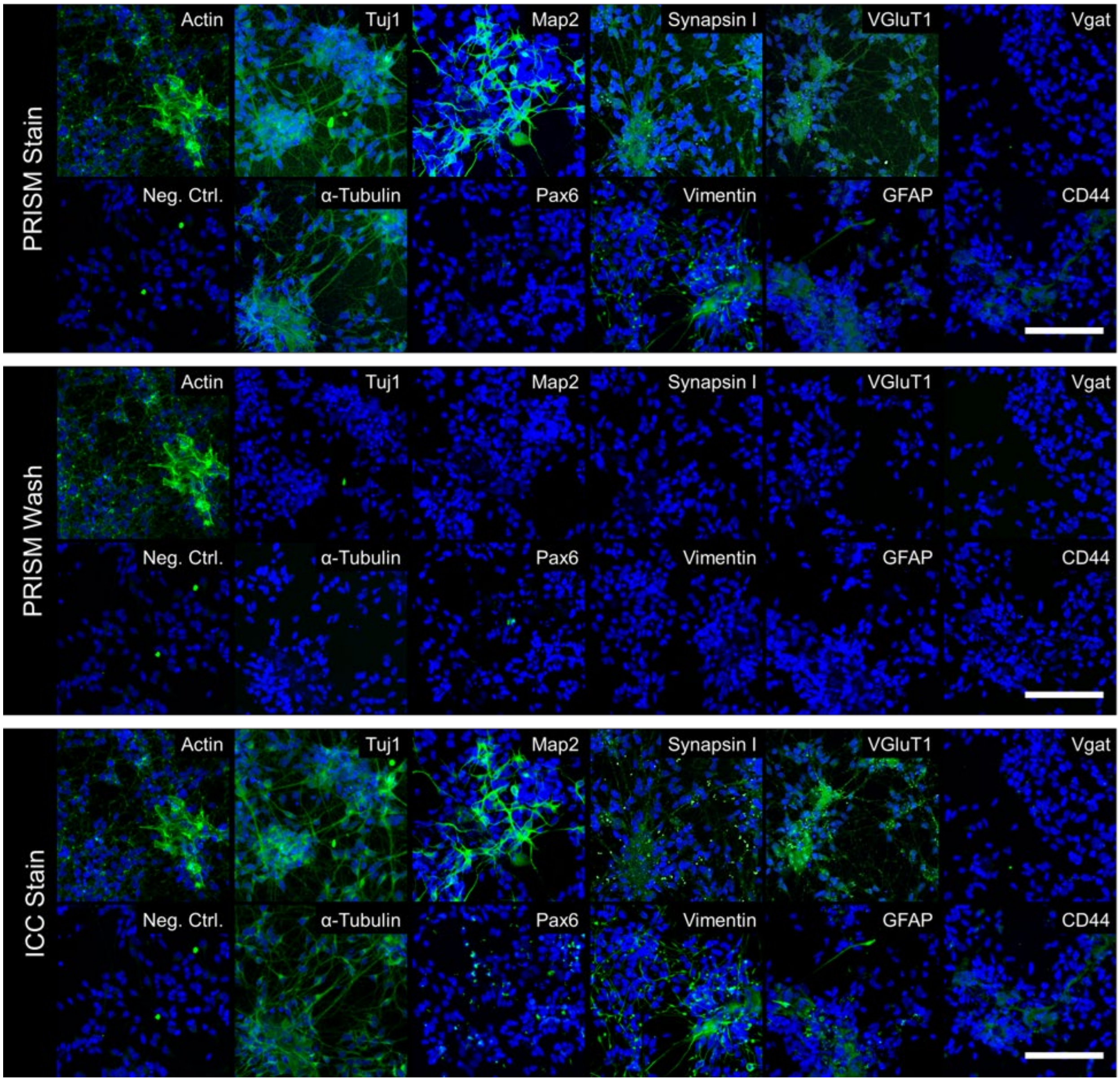

Supplementary Figure 7: Comparison between ICC staining and PRISM staining in human iPSC-derived cortical neurons, dissociated into 2D culture and maintained for 14 *days in vitro*. Representative IF staining with the PRISM characterization panel using the full selection of cortical markers. Comparing traditional antibody-antibody staining (ICC Stain) versus our conjugated antibody platform (PRISM Stain), while residual signal left after the low salt buffer wash is shown in the (Post-Stain) panel for each conjugated antibody. To generate the control ICC signal, each culture was first stained with the PRSIM antibodies, nine positions were marked and imaged for each marker. The cultures were then washed and stained with regular secondary antibodies against the primary, then the same areas were imaged again. Representative images shown in this figure are from 6 different staining runs. Pax 6 staining with the PRISM antibody was subject to sensitivity issue. Scale bars are 100 microns.

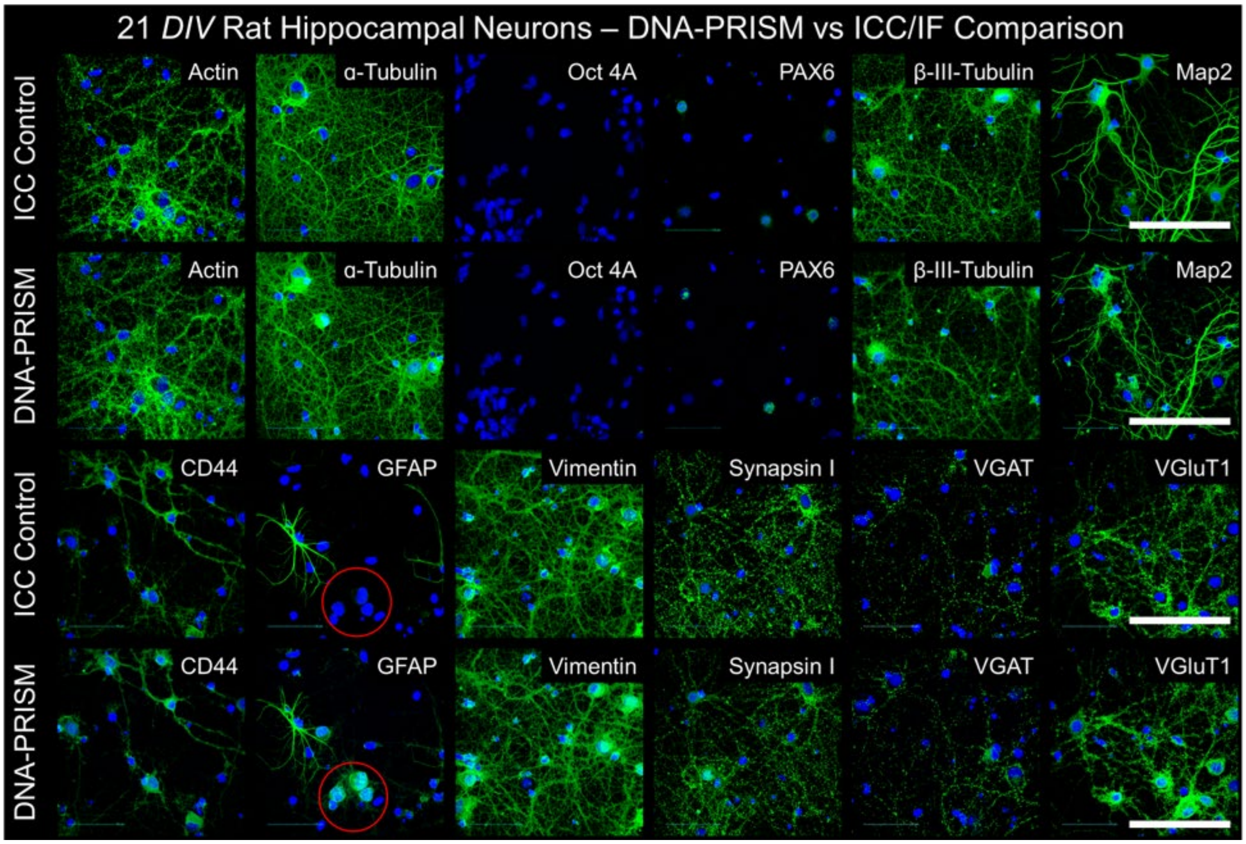

Supplementary Figure 8: Comparison between ICC staining and DNA-PRISM staining in rat hippocampal neurons, dissociated into 2D culture and maintained for 21 *days in vitro* (DIV). Representative immunofluorescent staining with the PRISM characterization panel using the full selection of cortical markers. Comparing traditional antibody-antibody staining (ICC Control) versus our conjugated antibody platform (DNA-PRISM) suggests very high correlation between the readouts in all, but the GFAP marker, where there is a subset of aberrant staining artefacts (circled in red). This might be due to this specific antibody not being validated to work in rat dissociated cultures. Importantly, the staining patterns for human (shown in this paper) and mouse (data not shown) cortical neurons, which are both validated by the manufacturer, are almost identical between control ICC and our PRISM readouts. Representative data shown in this figure from 4 independent staining runs. Scale bars are 100 microns.

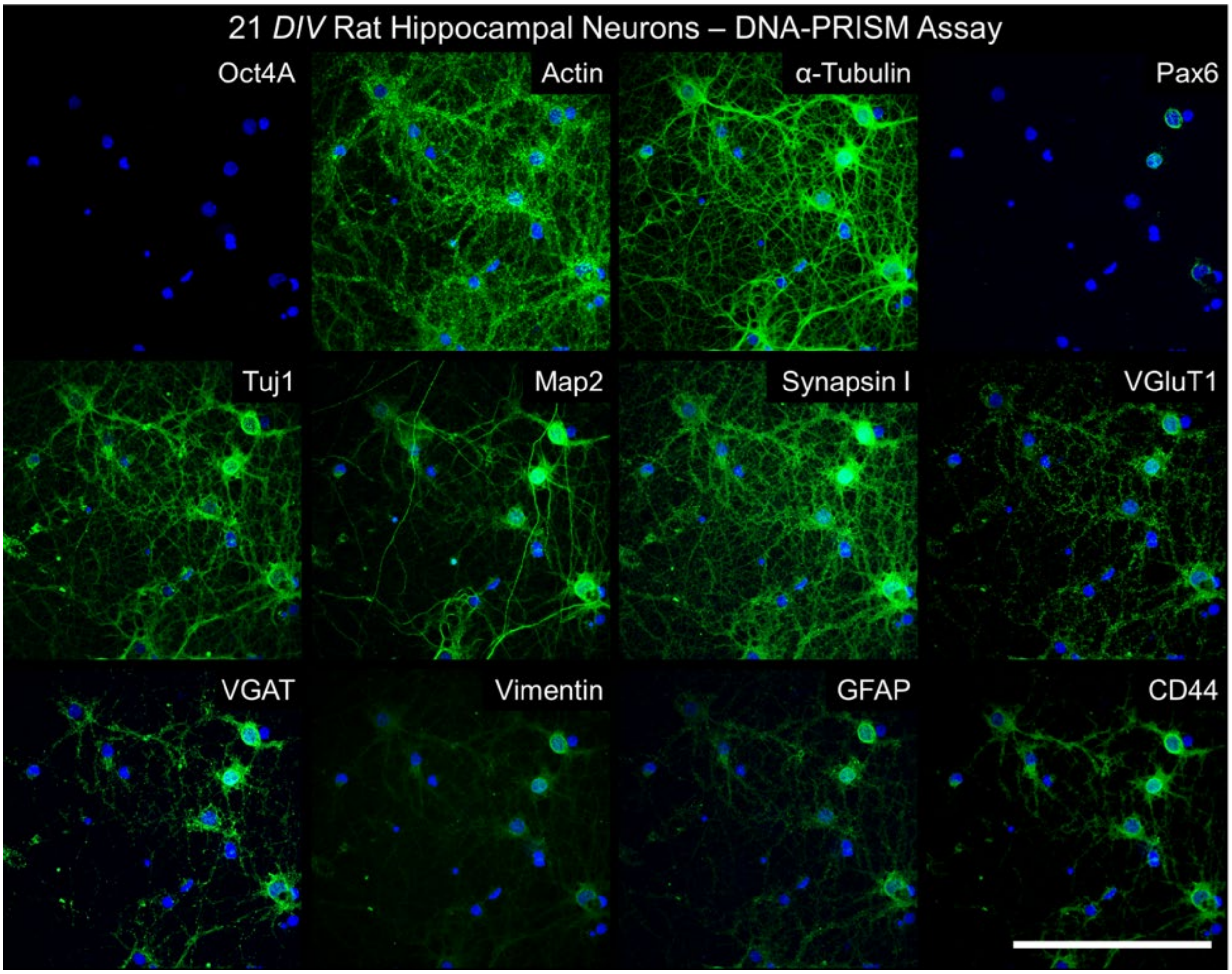

Supplementary Figure 9: DNA-PRISM antibodies can generate high-content data in 2D dissociated rat hippocampal neurons cultures. Representative immunofluorescent staining with the PRISM characterization panel using the full selection of cortical markers and DNA-based imager strands. The GFAP and Vimentin markers in our panel were selected and optimized to work in human cortical cultures, so they exhibit some staining aberrations when used in rat cultures. Scale bars are 100 microns.

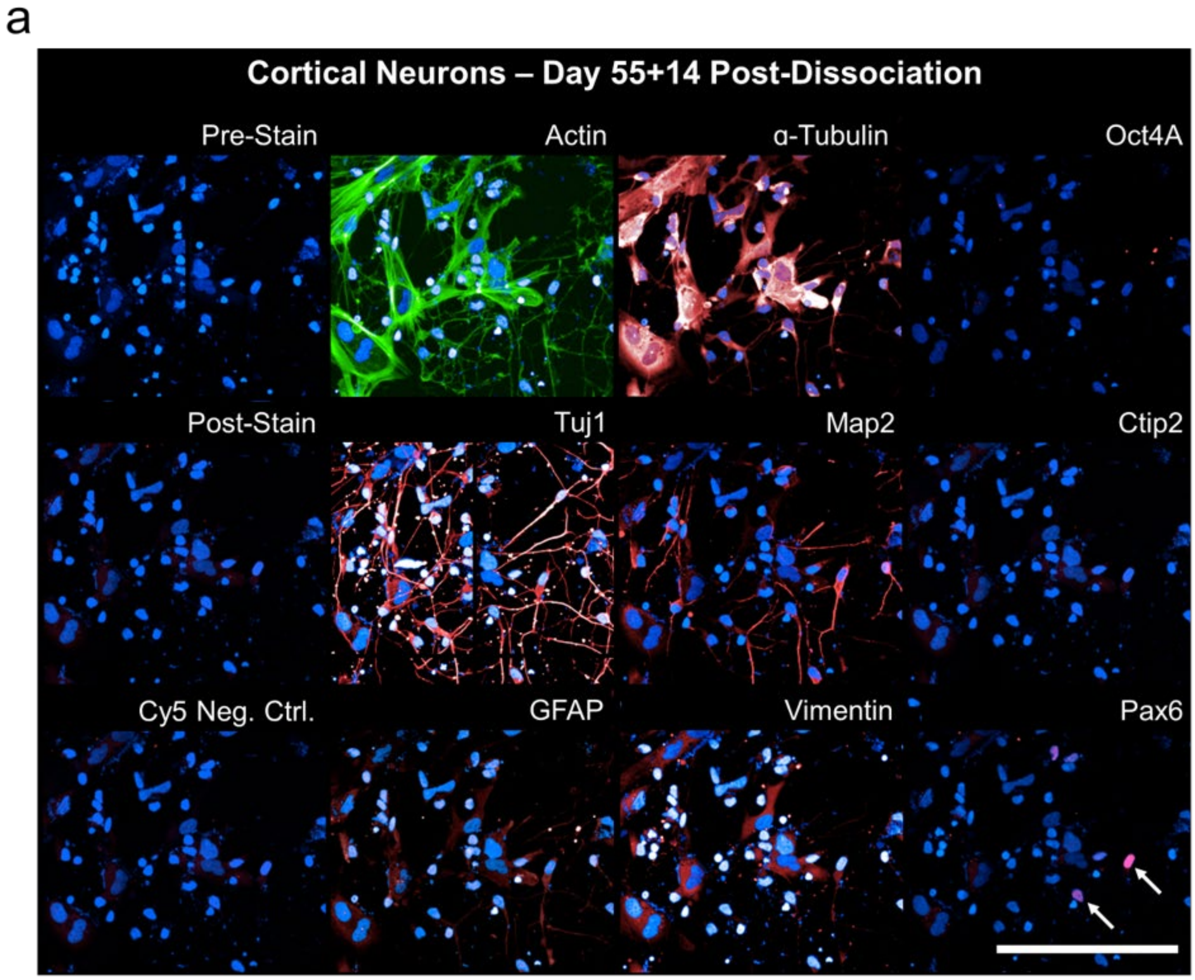

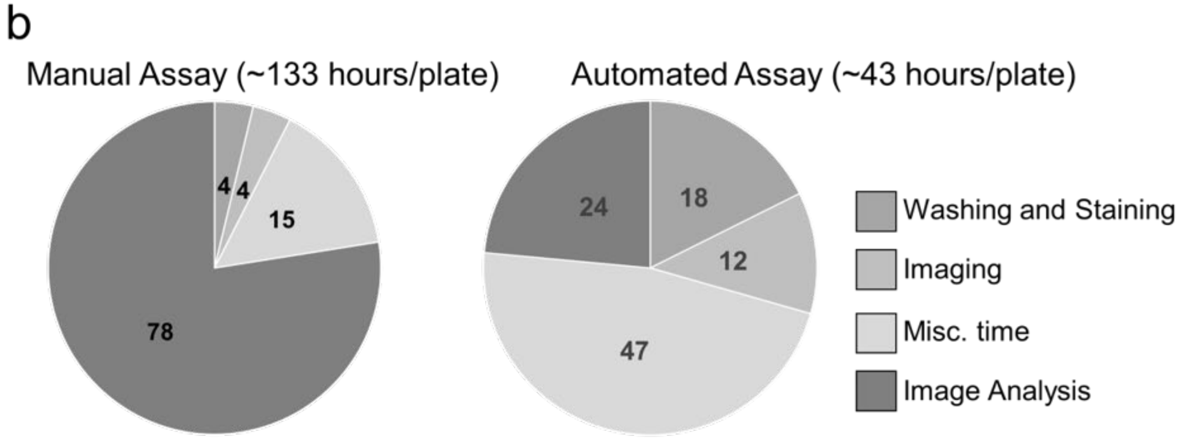

Supplementary Figure 10: Automated PRISM staining and imaging generates comparable outputs to manual assays, with a significant increase of the depth of analysis and a decrease in required run times (a) Representative images generated in cortical neural cultures using an abridged PRISM panel on the PerkinElmer Phenix. **(b)** Comparison of percent time spent between a representative manual assay analysis versus an automated pipeline for a single 96-well plate. Automating the PRISM assay is on average three times faster compared to a manual assay. Scale bars is 100 microns.

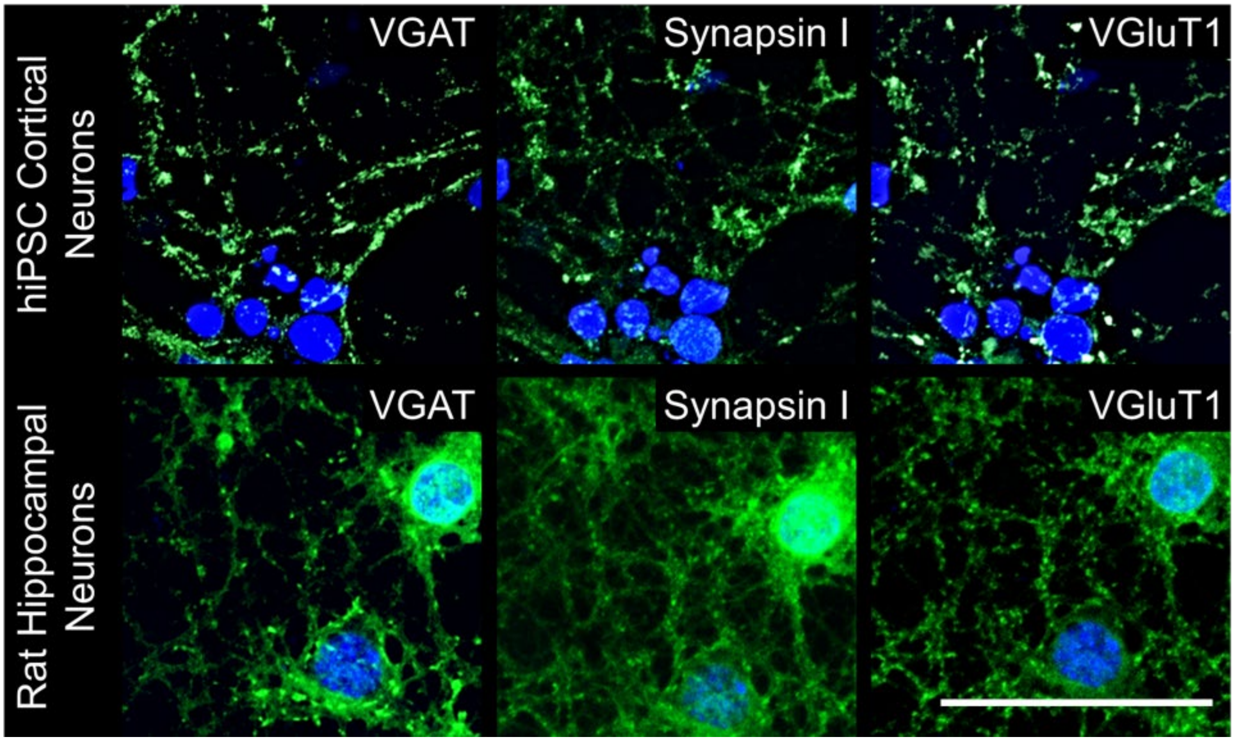

Supplementary Figure 11: Zoom-in on synaptic markers stained with DNA-PRISM antibodies. Representative images of three synaptic markers stained in stem cell derived cortical neurons and in dissociated rat hippocampal neurons. Images were taken in the same field of view in cultures that were fixed at 14 days post-seeding. Scale bar is 25 microns.

GFAP	RnD Systems	AF2594	sheep	Yes	Yes
GFAP	Abcam	ab190288	mouse	Yes	No
Nestin	RnD Systems	MAB1259	mouse	Yes	Yes
Nestin	ThermoFisher	MA1-110	mouse	Yes	Yes
Nestin	Abcam	ab22035	mouse	Yes	Yes
Vimentin	RnD Systems	MAB2105	rat	Yes	Yes
Vimentin	RnD Systems	AF2105	goat	Yes	No
Vimentin [V9]	ThermoFisher	MA5-11883	mouse	Yes	Yes
Vimentin	Abcam	ab92547	rabbit	Yes	Yes
CD44	RnD Systems	AF6127	sheep	Yes	No
CD44	Novus	NBP1-47386	mouse	Yes	Yes
CD44	Abcam	ab15707	rabbit	Yes	Yes
GLAST/EAAT1	Abcam	ab416	rabbit	Yes	Yes
S100-beta	Abcam	ab52642	rabbit	Yes	Yes
TBR1	Abcam	ab31940	rabbit	Yes	Yes
Ctip2	Abcam	ab18465	rat	Yes	Yes
MAP2	Novus	NB300-213	chicken	Yes	Yes
MAP2	Novus	NB100-98717	sheep	Yes	No
β-III-Tubulin/Tuj1	Sigma Aldrich	T5076-200ul	mouse	Yes	Yes
β-III-Tubulin/Tuj1	Novus	NB100-1612	chicken	Yes	No
SATB2	Abcam	ab51502	mouse	Yes	Yes
CDP/CUTL1/CUX1	Pierce Chemical	PA525788	rabbit	Yes	Yes
Synapsin I	Abcam	ab64581	goat	Yes	Yes
GABA B1 Receptor	Abcam	ab55051	mouse	Yes	Yes
200kD Neurofilament	Novus	AF3108	goat	Yes	No
200kD Neurofilament	Novus	NBP1-97726	mouse	Yes	Yes
CaMKII-alpha	Abcam	ab54925	mouse	Yes	Maybe
VGluT1	ThermoFisher	48-2400	rabbit	Yes	No
Vgat	Novus	MAB6847	mouse	Yes	Yes
Gephyrin	Abcam	ab181382	rabbit	Yes	Maybe
Islet 1	RnD Systems	AF1837	goat	Yes	Yes
Islet 1	Abcam	ab109517	rabbit	Yes	Yes
Cortactin	Novus	NBP2-15971	rabbit	Yes	Maybe
ChAT	Abcam	ab181023	rabbit	Yes	Yes
Pax6	ThermoFisher	42-6600	rabbit	Yes	Yes
SOX2	RnD Systems	MAB2018	mouse	Yes	Yes
ZO-1	ThermoFisher	61-7300	rabbit	Yes	Yes
SSEA1	RnD Systems	MAB2155	mouse	Yes	Yes
SOX1	Abcam	ab87775	rabbit	Yes	Yes
Nanog	ThermoFisher	PA1-097	rabbit	Yes	Yes
E-Cadherin	ThermoFisher	13-1700	mouse	Yes	Yes
SSEA4	ThermoFisher	MA1-021	mouse	Yes	Yes
Oct-3/4A	RnD Systems	MAB1759	rat	Yes	Yes
α-Tubulin [TU-01]	ThermoFisher	MA1-19162	mouse	Yes	Yes
α-Tubulin [TU-01]	ThermoFisher	13-8000	mouse	Yes	Yes
α-Tubulin [DM1a]	Sigma Aldrich	T6199-200ul	mouse	Yes	No
α-Tubulin [DM1a]	Abcam	ab7291	mouse	Yes	Yes
α-Tubulin [DM1a]	Novus	NB100-690	mouse	Yes	Yes
γ-Tubulin	Abcam	ab11316	mouse	Yes	Yes
Pericentrin	Abcam	ab4448	rabbit	Yes	Yes
Actin/Phalloidin488	ThermoFisher	A12379	-	Yes	Yes
Actin/Phalloidin568	ThermoFisher	A12380	-	Yes	Yes
Actin/Phalloidin598	ThermoFisher	A12381	-	Yes	Yes
Actin/Phalloidin660	ThermoFisher	A22285	-	Yes	Yes
Donkey anti-Guinea Pig	Jackson	706-005-148	-	Yes	Yes
Donkey anti-Chicken	ThermoFisher	SA1-72002	-	Yes	Yes
Donkey anti-Chicken	Rockland	603-701-C37	-	Yes	Yes
Donkey anti-Mouse	ThermoFisher	A16019	-	Yes	Yes
Donkey anti-Rabbit	ThermoFisher	A16037	-	Yes	Yes
Donkey anti-Goat	ThermoFisher	A16007	-	Yes	Yes
Donkey anti-Rat	ThermoFisher	A24545	-	Yes	Yes
Donkey anti-Sheep	ThermoFisher	A16050	-	Yes	Yes
DAPI	ThermoFisher	62248	-	Yes	Yes

Supplementary Table 1: Full list of antibodies tested and validated for compatibility with the necessary modifications to generate PRISM antibodies. Candidate markers that still maintained their specific recognition sites and staining pattern when compared to the unmodified version are marked in white, while markers that were inconsistent between conjugation runs are marked with yellow, and markers that we were unable to convert into PRISM antibodies successfully are marked in red.

Marker	iPSCs	Human NPCs	Human Neurons	Human Glial Cells	Human Radial Glia	Early Astrocytes	Late Astrocytes
MAP2	-	-	+	-	-	-	-
β-III-Tubulin	-	-	+	-	-	-	-
Vgat	-	-	+	-/+	-	-	-/+
VGluT1	-	-	+	-/+	-	-	-/+
Synapsin I	-	-	+	-/+	-	-	-/+
Vimentin	-	-	-	+	+	+	
GFAP	-	-	-	+		-	+
CD44				+			+
PAX6	-	+	-		+	-	-
Oct4A	+	-	-	-	-	-	-
α-Tubulin	+	+	+	+	+	+	+
Actin	+	+	+	+	+	+	+
Hoechst	+	+	+	+	+	+	+

Supplementary Table 2: List of PRISM antibodies that allow for extensive and parallel neural culture characterization. Curated panel that is able to analyze complex cortical and motor neuron cultures, both as healthy controls as well as in disease modeling, while also maintaining the flexibility to evaluate other neural culture types and states with minimal modifications. Human glia cells could be seen as expressing synaptic markers (-/+), due to their role in harvesting and recycling these in neural co-culture

PRISM Staining Protocol	
**1. Fixation**	4% Paraformaldehyde in 1xPBS for 15min at room temperature (RT)
**2. Paraformaldehyde Quench**	100mM Glycine in ddH$_2$O for 10min at RT
**3. Permeabilization**	0.2% TritonX-100 in 1xPBS for 15min at RT
**4. Wash**	2x5min with 1xPBS at RT
**5. Blocking I**	1x PBS + 1:100 RNase A1 and RNase T1 Cocktail for 30min at RT
**6. Blocking II**	2% BSA in 1xPBS (w/v) + 1mg/ml salmon sperm DNA for 1 hour at 4°C
**7. Wash**	2x5min with 1xPBS at room temperature
**8. Primary Antibody Addition**	Primary antibodies added at 1:200 concentration & incubated at 4°C o/n
**9. Wash**	2x5min with 1xPBS at room temperature
**10. PRISM Secondary Antibodies Addition**	Secondary conjugated antibodies added at 1:200 concentration and incubated at 4°C overnight
**11. Wash**	2x5min with PRISM Wash Buffer at RT
**12. PRISM Primary Antibodies Addition**	Primary conjugated antibodies added at 1:100 concentration and incubated at 4°C overnight
**13. Wash**	2x5min with PRISM Wash Buffer at RT
**14. Nuclear & Phalloidin Staining**	1:200 DAPI (1mg/ml) and 1:200 Ph488 Added and incubated for 1 hour at RT
**15. Wash**	2x5min with 1xPBS at RT and image as soon as possible
**16. Add a PRISM Imaging Strand**	Add 10-20nM imager strand in Imaging Buffer and incubate at RT for 10min.
**17. Image Sample**	Wash cells once in Imaging Buffer, keep them in this buffer and image sample
**18. Wash**	3x5min incubation in Wash Buffer at RT
**19. Cycle PRISM Imaging**	Cycle through steps 15-17 until all markers are imaged
**20. Data Analysis**	Post-Acquisition processing and analysis in CellProfiler and/or FIJI

DNA-PRISM-Specific Buffers

Blocking Buffer (1xPBS + 2% BSA; v/w)
- 50ml 1xPBS
- 1g BSA
- 500ul 100x pen/strep
- Store at 4°C
- **Just before blocking:**
  - Add ssDNA to 1mg/ml final conc.

Imaging Buffer (500mM NaCl in 1xPBS)
- 50mL of 1xPBS
- 1.46g of NaCl
- **Adjust pH to ~8.0**
- Store at room temperature

Wash Buffer (0.01x PBS)
- 50mL of ddH2O
- 500µL of 1x PBS
- Store at room temperature

Supplementary Table 3: Detailed outline on DNA-PRISM staining protocol. Steps and explanations starting from cell fixation all the way to the post-acquisition data processing, including the PRISM-specific buffers that are required to cycle imager strands to generate the 12 marker neural panel images.

Cortical Neuron Differentiation Protocol

Protocol Step	Step Details	Notes
Adapt iPSCs for Cortical Differentiation	Seed single cell dissociated iPSCs into a spinner flask in mTeSR1 media supplemented with 10µM ROCKi for 2 days.	Dissociate iPSC colonies with UltraPure 500µM EDTA for 10min at 37°C.
mTeSR1 NPC Adaptation	Supplement mTeSR1 media with SB (10µM), LDN (1µM), and XAV939 (2µM) small molecules for 1 day.	Full media change. Prepare the mTeSR1-based differentiation media immediately prior to use.
KSR Pulse & Neural Patterning	Supplement KSR Pulse media with SB (10µM), LDN (1µM), and XAV939 (2µM) small molecules for 3 days.	Do one full media change after 24 hours, then keep cells in KSR Pulse media for 48 hours.
cNIM Adaptation & Neural Patterning I	Mix KSR Pulse media with cNIM media at 75/25 and supplement it with SB (10µM) and LDN (1µM). Incubate for 2 days.	Full media changes every 2 days. Prepare media mix immediately prior to use.
cNIM Adaptation & Neural Patterning II	Mix KSR Pulse media with cNIM media at 50/50 and supplement it with SB (10µM) and LDN (1µM). Incubate for 2 days.	Full media changes every 2 days. Prepare media mix immediately prior to use.
cNIM Adaptation & Neural Patterning III	Mix KSR Pulse media with cNIM media at 25/75 and supplement it with SB (10µM) and LDN (1µM). Incubate for 2 days.	Full media changes every 2 days. Prepare media mix immediately prior to use.
cNIM Cortical Differentiation	Incubate cultures in cNIM media for 9 days.	Full media changes every 3 days. Full cNIM media can be stored at 4°C for the duration.
NB Cortical Differentiation	Incubate cultures in NB media supplemented with BDNF (10ng/mL) and GDNF (10ng/mL) for 20+ days.	Full media changes every 4 days. Full NB media can be stored at 4°C for the duration.
Cortical Neurons QC & Cryo-Storage	Dissociate cortical spheroids at Day 41+ and freeze down at ~20 million cells/mL, OR use generated cells for downstream applications.	Dissociate spheroids in Trypsin-EDTA for 20min at RT, count cells and record viability.
Cortical Neuron 2D Culture Plating	Seed cortical neurons on poly-D-Lysine (25µg/mL), poly-L-ornithine (25µg/mL), Fibronectin (10µg/mL), and Laminin (10µg/mL) coated plates in NB+BDNF/GDNF (10ng/mL), OR BrainPhys+BDNF/GDNF (20ng/mL) media.	Dilute PDL and PLO in 1X Borate in water. Add 10µM ROCKi to the NB+BDNF/GDNF media and do 50% media change 24 hours later.
NB Cortical Maturation II	Maintain 2D cortical cultures in NB+BDNF/GDNF (10ng/mL), OR BrainPhys+BDNF/GDNF (20ng/mL) for 1 week common marker analysis, and for 2+ weeks for synapse formation.	50% media changes every 4 days; if keeping long-term cultures, add Laminin (10µg/mL) in the cortical media used once a week.

Supplementary Table 4: Protocol steps, including expanded step details and notes for cortical neuron differentiation using human iPSCs. Detailed notes include small molecule concentrations, used in this protocol, as well as comments that improve the efficacy of each step in this complex differentiation.

Motor Neuron Differentiation Protocol		
**Protocol Step**	**Step Details**	**Notes**
Pluripotent iPSCs Single Cell Dissociation	Day -2: Use UltraPure EDTA (500µM) for 10min at 37°C.	Different lines may require adjustment on dissociation time with EDTA.
EB/Spheroid Formation	Day -2-0: Seed cells in ultra-low attachment plates in mTESR1 media supplemented with ROCKi (10µM and FGF-2 (20ng/mL).	Keep forming EBs in this media for 24 hours, then replace with fresh mTESR1 media for 24 hours.
Differentiate Spheroids in Motor Neuron Differentiation (MND) Media	Day 0-2: MND media supplemented with SB431542 (10µM) and LDN193189 (100nM), plus CHIR99021 (3µM).	Collect spheroids in a conical tube and spin them down for 5min at 500rpm before media change.
Differentiate Spheroids in Motor Neuron Differentiation (MND) Media	Day 2-4: MND media supplemented with SB431542 (10µM) and LDN193189 (100nM), plus CHIR99021 (3µM), RA (1µM) and SAG (1µM).	Collect spheroids in a conical tube and spin them down for 5min at 500rpm before media change.
Differentiate Spheroids in Motor Neuron Differentiation (MND) Media	Day 4-6: MND media supplemented with SB431542 (10µM) and LDN193189 (100nM), plus RA (1µM) and SAG (1µM).	Collect spheroids in a conical tube and spin them down for 5min at 500rpm before media change.
Differentiate Spheroids in Motor Neuron Differentiation (MND) Media	Day 6-8: MND media supplemented with RA (1µM) and SAG (1µM).	Collect spheroids in a conical tube and spin them down for 5min at 500rpm before media change.
Differentiate Spheroids in Motor Neuron Differentiation (MND) Media	Day 8-16: MND media supplemented with RA (1µM) and SAG (1µM), supplemented with DAPT (10µM), BDNF and GDNF (20ng/mL each). **50% media changes every two days**	Collect spheroids in a conical tube and spin them down for 5min at 500rpm before media change.
Motor Neuron Spheroids Dissociation to Single Cells	Day 16: EBs were then dissociated with a solution 0.25% Trypsin-EDTA and DNAse (25µg/mL) until single cells; ~15min at RT.	Collect spheroids in a conical tube and spin them down for 5min at 500rpm before media change.
Replate Motor Neurons in Motor Neuron Maturation (MNM) Media	Day 16: Seed cells on poly-D-Lycine (25µg/mL), poly-L-ornithine (25µg/mL), Fibronectin (10µg/mL), and Laminin (10µg/mL) coated plates in MNM media plus BDNF and GDNF (20ng/mL each).	Coat plates o/n with 1X Borate in water plus poly-L-ornithine (25µg/mL), wash 3x with PBS, then coat o/n with 1xPBS plus Fibronectin & Laminin (10µg/mL each).
Mature Motor Neurons in Motor Neuron Maturation (MNM) Media	Day 16-30: Grow cells in MNM media plus BDNF and GDNF (20ng/mL each). Add Laminin (10µg/mL) once a week for long-term cultures. **50% media changes twice a week**	Motor neurons can be fixed for evaluation after 6 days of maturation, but for synaptic formation, 14 days in MNM media is recommended.

Supplementary Table 5: Protocol steps, including expanded step details and notes for motor neuron differentiation using human iPSCs. Detailed notes include small molecule concentrations, used in this protocol, as well as comments that improve the efficacy of each step in this complex differentiation.

Astrocyte Differentiation Protocol		
Protocol Step	Step Details	Notes
Dissociate and Plate iPSCs as Single Cells on TC-Treated Plates	Seed cells at ~40% confluency in mTESR1 media supplemented with 10μM ROCKi overnight.	Coat plates with 1:100 Matrigel for 2 hours at 37°C.
Expand iPSCs until 60% Confluent	Full media change with mTESR1 without ROCKi.	Cells are usually ~60% confluent in two days.
Neural Progenitor Cell Differentiation (NPCs)	Change media to Neural Induction Media (NIM). Full media changes every day until cells are fully confluent.	Cells are usually ~90% confluent in two to three days.
Passage Confluent Cell Culture	Seed cells at ~40% confluency in fresh NIM media.	Use Trypsin-EDTA to dissociate cells, spin down at 500rpm for 5min and seed cells on Matrigel-coated plates.
NPC Differentiation	Use Neural Induction Media (NIM). Full media changes every day until cells are fully confluent.	Cells are usually ~90% confluent in three to five days.
Passage Confluent Cell Culture	Seed cells at ~40% confluency in fresh NIM media on Matrigel-coated plates.	Use Trypsin-EDTA to dissociate cells, spin down at 500rpm for 5min and seed cells on Matrigel-coated plates
Finish NPC Differentiation	Full media changes with NIM media every day until Day 14. Dividing NPC c ultures can be cryo-stored after Day 14.	Passage cells with Trypsin-EDTA to ~40% confluency every time they are ~90% confluent. Use Matrigel-coated plates.
Seed NPCs at ~30% Confiency on TC-Treated Plates for Astrocyte Differentiation	Change media to Astrocyte Media (AM; Sciencell). Full media changes every 3 days until cells are fully confluent.	Passage cells with Trypsin-EDTA to ~30% confluency every time they are ~90% confluent. Use Matrigel-coated plates.
Astrocyte Differentiation	Full media changes with AM media every 3 days until Day 21. Dividing astrocyte cultures can be cryo-stored after Day 21.	At day 21, differentiated cultures are mostly immature dividing astrocytes, expressing multiple canonical markers.

Supplementary Table 6: Protocol steps, including expanded step details and notes for astrocyte differentiation using human iPSCs. Detailed notes include small molecule concentrations, used in this protocol, as well as comments that improve the efficacy of each step in this differentiation.

LNA Sequence Name	Docker sequence	Imager sequence
P1	TTATACATCTA	T+AGAT+G+TATAA
P2	TTATCTACATA	TATGT+A+G+ATAA
P3	TTTCTTCATTA	TAAT+G+A+AGAAA
P4	TTATGAATCTA	TA+GAT+T+CATAA
P5	TTTTAGGTAAA	TT+T+A+CCTAAAA
P6	TTAATTGAGTA	T+A+C+TCAATTAA
P7	TTAATTAGGAT	A+T+CCT+AATTAA
P8	TTATAATGGAT	A+T+CC+ATTATAA
P9	TTTAATAAGGT	A+CC+T+TATTAAA
P10	TTATAGAGAAG	C+T+TC+TCTATAA
P12	TTATAGTGATT	A+ATC+A+CTATAA

DNA Sequence Name	Docker sequence	Imager sequence
M1	CATATATTCGT	GTATATAAGCA
M2	TCAGATTTTCA	AGTCTAAAAGT
M3	CTATCAGTTTA	GATAGTCAAAT
M4	TACTCATTGTA	ATGAGTAACAT
M5	TTTTTAAGCCA	AAAAATTCGGA
M6	TATTGCAATCT	ATAACGTTAGA
M7	GTTTTCATACA	CAAAAGTATGT
M8	CATCTTTATAG	GTAGAAATATC
M9	CTATAGTATCT	GATATCATAGA
M10	ACCATTTTATG	TGGTAAAATAC

*** A/T/G/C+ denotes LNA nucleotide**

Supplementary Table 7: Published LNA/DNA [11] and newly developed DNA-only PRISM pair sequences list. Docker strands are the ones that are conjugated to antibodies, while the imager strands are the ones that are used to generate the IF/ICC signal by having them conjugated to a fluorophore, ATTO647N in the case of the LNA/DNA imagers, or ATTO647N and ATTO590 in the case of DNA-only imagers. Pluses (+) after the respective nucleotides denote an LNA substitution within the oligo. Docker sequences are presented in the 5'-3' direction, while the imager sequences are shown in the 3'-5- direction in the table. Fluorophores were conjugated on the 5' ends of the imager strands.

Supplementary Data 1: Data enclosed includes the raw analysis files and images that were sued to generate the culture makeup of the day 55 stem cell derived cortical neurons. Included are also the criteria that were used to separate each cell type.

Supplementary Data 2: Data enclosed includes the raw analysis files and images that were sued to generate the culture makeup of the day 85 stem cell derived cortical neurons. Included are also the criteria that were used to separate each cell type.

Supplementary Data 3: CellPRofiler pipeline that was developed and used to characterize the PRISM imaging data generated in the paper.
