# Supplementary material for: Resolving cell state in iPSC-derived human neural samples with multiplexed fluorescence imaging
Source: Commun Biol. 2021 Jun 24;4:786. doi: 10.1038/s42003-021-02276-x (PMC8225800; doi:10.1038/s42003-021-02276-x)
Supplement: Supplementary file 3 — Description of Supplementary Files [file 42003_2021_2276_MOESM3_ESM.pdf]

Description of Additional Supplementary Files

File name: Supplementary data 1

Description: Data enclosed includes the raw analysis files and images that were sued to generate the culture makeup of the day 55 stem cell derived cortical neurons. Included are also the criteria that were used to separate each cell type.

File name: Supplementary data 2

Description: Data enclosed includes the raw analysis files and images that were sued to generate the culture makeup of the day 85 stem cell derived cortical neurons. Included are also the criteria that were used to separate each cell type.

File name: Supplementary data 3

Description: CellPRofiler pipeline that was developed and used to characterize the PRISM imaging data generated in the paper.
