# Supplementary material for: Resolving cell state in iPSC-derived human neural samples with multiplexed fluorescence imaging
Source: Commun Biol. 2021 Jun 24;4:786. doi: 10.1038/s42003-021-02276-x (PMC8225800; doi:10.1038/s42003-021-02276-x)
Supplement: Supplementary file 7 — Reporting Summary [file 42003_2021_2276_MOESM7_ESM.pdf]

nature research

Corresponding author(s): Mark Bathe
Lee Rubin

Last updated by author(s): May 5, 2021

Reporting Summary

Nature Research wishes to improve the reproducibility of the work that we publish. This form provides structure for consistency and transparency in reporting. For further information on Nature Research policies, see our Editorial Policies and the Editorial Policy Checklist.

Statistics

For all statistical analyses, confirm that the following items are present in the figure legend, table legend, main text, or Methods section.

n/a	Confirmed	
☐	☒	The exact sample size (n) for each experimental group/condition, given as a discrete number and unit of measurement
☐	☒	A statement on whether measurements were taken from distinct samples or whether the same sample was measured repeatedly
☐	☒	The statistical test(s) used AND whether they are one- or two-sided<br>*Only common tests should be described solely by name; describe more complex techniques in the Methods section.*
☐	☒	A description of all covariates tested
☐	☒	A description of any assumptions or corrections, such as tests of normality and adjustment for multiple comparisons
☐	☒	A full description of the statistical parameters including central tendency (e.g. means) or other basic estimates (e.g. regression coefficient) AND variation (e.g. standard deviation) or associated estimates of uncertainty (e.g. confidence intervals)
☐	☒	For null hypothesis testing, the test statistic (e.g. F, t, r) with confidence intervals, effect sizes, degrees of freedom and P value noted<br>*Give P values as exact values whenever suitable.*
☐	☒	For Bayesian analysis, information on the choice of priors and Markov chain Monte Carlo settings
☐	☒	For hierarchical and complex designs, identification of the appropriate level for tests and full reporting of outcomes
☐	☒	Estimates of effect sizes (e.g. Cohen's d, Pearson's r), indicating how they were calculated

Our web collection on statistics for biologists contains articles on many of the points above.

Software and code

Policy information about availability of computer code

Data collection	We used the software provided with the Opera Phenix (PerkinElmer) to aquire and process the generated control and DNA-PRISM images.
Data analysis	We used CellProfiler to analyze our DNA-PRISM images

For manuscripts utilizing custom algorithms or software that are central to the research but not yet described in published literature, software must be made available to editors and reviewers. We strongly encourage code deposition in a community repository (e.g. GitHub). See the Nature Research guidelines for submitting code & software for further information.

Data

Policy information about availability of data

All manuscripts must include a data availability statement. This statement should provide the following information, where applicable:

- Accession codes, unique identifiers, or web links for publicly available datasets
- A list of figures that have associated raw data
- A description of any restrictions on data availability

The data generated for this manuscript and/or used to support the findings of this study are presented in this manuscript and its supplementary files.

Field-specific reporting

Life sciences study design

All studies must disclose on these points even when the disclosure is negative.

Sample size	We analyzed all cells that were present in the images for each run. As such the sample sizes were were working with were the entire cell population that was present, which would be largest possible sample size for the given experiment.
Data exclusions	No data was excluded from each analysis.
Replication	We performed the DNA-PRISM staining for at least N=3 times on different cortical cultures, with representative images shown in the figures. All replicate were able to successfully be stained with the PRISM antibodies.
Randomization	Not relevant to the study, as we analyzed all generated data.
Blinding	The CellProfile pipeline was desgined to blindly allocate cell type, based on DNA-PRSIM staining. The rest of the experiments, that is, staining, antibody generation, and stem cell differentiation validation, optimization, and imaging prep did not need to be blinded.

Reporting for specific materials, systems and methods

We require information from authors about some types of materials, experimental systems and methods used in many studies. Here, indicate whether each material, system or method listed is relevant to your study. If you are not sure if a list item applies to your research, read the appropriate section before selecting a response.

Materials & experimental systems

n/a	Involved in the study	
☐	☒	Antibodies
☒	☐	Eukaryotic cell lines
☒	☐	Palaeontology and archaeology
☒	☐	Animals and other organisms
☒	☐	Human research participants
☒	☐	Clinical data
☒	☐	Dual use research of concern

Methods

n/a	Involved in the study	
☒	☐	ChIP-seq
☒	☐	Flow cytometry
☒	☐	MRI-based neuroimaging

Antibodies

Antibodies used

Marker; Company; Catalog #; Host; Tested; Working
GFAP RnD Systems AF2594 sheep Yes Yes
GFAP Abcam ab190288 mouse Yes No
Nestin RnD Systems MAB1259 mouse Yes Yes
Nestin ThermoFisher MA1-110 mouse Yes Yes
Nestin Abcam ab22035 mouse Yes Yes
Vimentin RnD Systems MAB2105 rat Yes Yes
Vimentin RnD Systems AF2105 goat Yes No
Vimentin [V9] ThermoFisher MA5-11883 mouse Yes Yes
Vimentin Abcam ab92547 rabbit Yes Yes
CD44 RnD Systems AF6127 sheep Yes No
CD44 Novus NBP1-47386 mouse Yes Yes
CD44 Abcam ab15707 rabbit Yes Yes
GLAST/EAAT1 Abcam ab416 rabbit Yes Yes
S100-beta Abcam ab52642 rabbit Yes Yes
TBR1 Abcam ab31940 rabbit Yes Yes
Ctip2 Abcam ab18465 rat Yes Yes
MAP2 Novus NB300-213 chicken Yes Yes
MAP2 Novus NB100-98717 sheep Yes No
β-III-Tubulin/Tuj1 Sigma Aldrich T5076-200ul mouse Yes Yes
β-III-Tubulin/Tuj1 Novus NB100-1612 chicken Yes No
SATB2 Abcam ab51502 mouse Yes Yes
CDP/CUTL1/CUX1 Pierce Chemical PA525788 rabbit Yes Yes
Synapsin I Abcam ab64581 goat Yes Yes
GABA B1 Receptor Abcam ab55051 mouse Yes Yes
200kD Neurofilament Novus AF3108 goat Yes No
200kD Neurofilament Novus NBP1-97726 mouse Yes Yes
CaMKII-alpha Abcam ab54925 mouse Yes Maybe
VGluT1 ThermoFisher 48-2400 rabbit Yes No

Vgat Novus MAB6847 mouse Yes Yes
Gephyrin Abcam ab181382 rabbit Yes Maybe
Islet 1 RnD Systems AF1837 goat Yes Yes
Islet 1 Abcam ab109517 rabbit Yes Yes
Cortactin Novus NBP2-15971 rabbit Yes Maybe
ChAT Abcam ab181023 rabbit Yes Yes
Pax6 ThermoFisher 42-6600 rabbit Yes Yes
SOX2 RnD Systems MAB2018 mouse Yes Yes
ZO-1 ThermoFisher 61-7300 rabbit Yes Yes
SSEA1 RnD Systems MAB2155 mouse Yes Yes
SOX1 Abcam ab87775 rabbit Yes Yes
Nanog ThermoFisher PA1-097 rabbit Yes Yes
E-Cadherin ThermoFisher 13-1700 mouse Yes Yes
SSEA4 ThermoFisher MA1-021 mouse Yes Yes
Oct-3/4A RnD Systems MAB1759 rat Yes Yes
α-Tubulin [TU-01] ThermoFisher MA1-19162 mouse Yes Yes
α-Tubulin [TU-01] ThermoFisher 13-8000 mouse Yes Yes
α-Tubulin [DM1a] Sigma Aldrich T6199-200ul mouse Yes No
α-Tubulin [DM1a] Abcam ab7291 mouse Yes Yes
α-Tubulin [DM1a] Novus NB100-690 mouse Yes Yes
γ-Tubulin Abcam ab11316 mouse Yes Yes
Pericentrin Abcam ab4448 rabbit Yes Yes
Actin/Phalloidin488 ThermoFisher A12379 - Yes Yes
Actin/Phalloidin568 ThermoFisher A12380 - Yes Yes
Actin/Phalloidin598 ThermoFisher A12381 - Yes Yes
Actin/Phalloidin660 ThermoFisher A22285 - Yes Yes
Donkey anti-Guinea Pig Jackson 706-005-148 - Yes Yes
Donkey anti-Chicken ThermoFisher SA1-72002 - Yes Yes
Donkey anti-Chicken Rockland 603-701-C37 - Yes Yes
Donkey anti-Mouse ThermoFisher A16019 - Yes Yes
Donkey anti-Rabbit ThermoFisher A16037 - Yes Yes
Donkey anti-Goat ThermoFisher A16007 - Yes Yes
Donkey anti-Rat ThermoFisher A24545 - Yes Yes
Donkey anti-Sheep ThermoFisher A16050 - Yes Yes
DAPI ThermoFisher 62248 - Yes Yes

Validation

We validated each antibody based on positive vs negative and secondary-only staining. Then the generated imaged were compared to published examples and images from the manufacturer for the staining pattern. Only antibodies that generated matching results in our stem cell derived neural cultures were selected for DNA-PRISM modification and use.
